# The Effects of Bisphosphonates Used in Osteoporosis Treatment on Breast Cancer: Analysis with Integrative Bioinformatics Methods, DFT, ADMET and Molecular Docking Analysis

**DOI:** 10.3390/biology15120952

**Published:** 2026-06-18

**Authors:** Sevil Ceyhan Dogan, Kenan Goren

**Affiliations:** 1 Department of Physical Medicine and Rehabilitation, Faculty of Medicine, Sivas Cumhuriyet University, Sivas 58140, Turkey; sevilceyhan@cumhuriyet.edu.tr; 2 Department of Organic Chemistry, Faculty of Arts and Sciences, Kafkas University, Kars 36100, Turkey

**Keywords:** bisphosphonates, breast cancer, osteoporosis, bioinformatics, molecular docking, DFT, ADMET, gene expression, survival analysis

## Abstract

This study evaluated the structural, electronic, pharmacokinetic, and receptor-binding properties of three bisphosphonate derivatives, alendronate, risedronate, and zoledronate, to investigate their therapeutic relevance in osteoporosis and breast cancer. Density Functional Theory (DFT) calculations at the B3LYP/6-31G(d,p) level showed that risedronate exhibited the highest kinetic stability (ΔE = 6.7468 eV), whereas zoledronate displayed greater chemical reactivity (ΔE = 2.9669 eV) and the strongest nonlinear optical response (β = 1.20 × 10^−30^ esu). ADMET analysis indicated acceptable safety profiles for all compounds, although high polarity and low lipophilicity may limit oral bioavailability. Molecular docking against 11 breast cancer- and bone metabolism-related targets revealed favorable binding affinities, particularly for zoledronate and risedronate. Zoledronate showed strong interactions with *ESR2*, *VEGFR*/*KDR*, *GGPS1*, and *FPPS*, whereas risedronate exhibited notable affinity for *BRCA2* and *MMP9*. Bioinformatics analyses identified significant dysregulation of *GGPS1*, *FDPS*, *TNFSF11*, *ESR1*, *MMP9*, and *BRCA2* in breast cancer tissues, while survival analysis linked elevated FDPS, *MMP9*, and *BRCA2* expression to poor prognosis. Network analyses highlighted pathways related to mevalonate metabolism, hormone signaling, angiogenesis, extracellular matrix remodeling, and the *RANK*/*RANKL*/*OPG* axis. These findings support the potential repurposing of bisphosphonates, particularly zoledronate, for breast cancer-associated bone disease.

## Simple Summary

Breast cancer can cause serious bone complications that reduce quality of life and increase fracture risk. Bisphosphonates are widely used to protect bone health, but their potential role in cancer treatment is not fully understood. In this study, we evaluated alendronate, risedronate, and zoledronate using computer-based methods to examine their properties, safety, and interactions with proteins involved in breast cancer and bone disease. The results showed that all three compounds may affect important biological targets associated with tumor growth and bone metabolism, with zoledronate showing the most promising overall performance. These findings suggest that bisphosphonates may offer benefits beyond bone protection and could help manage breast cancer-related bone disease. Further experimental studies are needed to confirm these results.

## 1. Introduction

Breast cancer remains one of the leading causes of cancer-related morbidity and mortality among women worldwide and is frequently associated with skeletal complications, including bone remodeling imbalance and osteoptosis-related alterations [1]. The complex interplay between breast cancer progression and bone metabolism has attracted considerable scientific interest, particularly due to the high incidence of bone-associated pathological conditions during disease progression [2]. Osteoptosis, characterized by dysregulated bone homeostasis and increased osteoclastic activity, has emerged as an important biological process contributing to skeletal deterioration and tumor-associated bone complications [3]. Therefore, identifying molecular targets involved in both osteoptosis and breast cancer progression is of substantial therapeutic significance [4].

Several proteins have been implicated in the regulation of breast cancer development, metastasis, angiogenesis, drug resistance, and bone microenvironment remodeling [5]. Estrogen receptors, including estrogen receptor alpha (*ER-α*/*ESR1*) and estrogen receptor beta (*ER-β*/*ESR2*), play pivotal roles in hormone-dependent breast cancer progression by regulating cellular proliferation, differentiation, and survival [6,7]. Similarly, *BRCA2*, a critical tumor suppressor gene involved in DNA repair pathways, contributes significantly to genomic stability, while its dysfunction is strongly associated with increased breast cancer susceptibility [8]. Matrix metalloproteinase-9 (*MMP-9*) has also been reported to facilitate extracellular matrix degradation, thereby promoting tumor invasion and metastatic dissemination [9]. Moreover, recent comprehensive meta-analyses have reaffirmed that tumoral overexpression of *MMP9* is a robust prognostic biomarker associated with increased lymph node metastasis, higher clinical staging, and shorter overall survival in breast cancer patients [10]. Moreover, ATP-binding cassette subfamily B member 1 (*ABCB1*/*MDR1*) is closely associated with multidrug resistance, a major challenge in breast cancer chemotherapy, whereas vascular endothelial growth factor receptor (*VEGFR*/*KDR*) is a key mediator of angiogenesis and tumor vascularization [11,12]. The molecular mechanisms of breast cancer and bone metastasis are highly complex, and recent reviews have further elucidated how the tumor microenvironment relies heavily on the *RANK*/*RANKL*/*OPG* signaling axis to coordinate osteoclast-mediated bone destruction [13]. In the context of bone metabolism, collagen type XVIII alpha 1 chain (*COL18A1*), osteoprotegerin (*OPG*/*TNFRSF11B*), and receptor activator of nuclear factor kappa-B ligand (*RANKL*/*TNFRSF11*) are recognized as essential regulators of bone remodeling and osteoclastogenesis [14,15,16]. Recent literature strongly highlights the mevalonate pathway not only as a crucial hub for cellular homeostasis but also as a promising metabolic target in cancer therapy [17]. The inhibition of this pathway by amino-bisphosphonates, which blocks the synthesis of essential isoprenoids, continues to show significant potential for precision therapy in breast cancer biology [18]. Particularly, the *OPG*/*RANKL* signaling axis plays a crucial role in maintaining bone integrity and has been implicated in osteoptosis-associated mechanisms [16]. Furthermore, geranylgeranyl diphosphate synthase 1 (*GGPS1*) and farnesyl pyrophosphate synthase (*FPPS*), enzymes involved in the mevalonate pathway, are important molecular targets of bisphosphonates and are closely associated with osteoclast inhibition and antiresorptive activity [19,20].

Bisphosphonates, including alendronate, risedronate, and zoledronate, are widely used therapeutic agents for the management of bone-related disorders due to their potent antiresorptive properties [21]. Recent studies have suggested that these compounds may also exhibit antitumor potential through modulation of apoptosis, angiogenesis, and metastatic signaling pathways [22]. In this regard, molecular docking analysis represents a valuable computational approach for evaluating ligand–protein interactions and predicting binding affinities at the molecular level [23].

In recent years, computational chemistry and in silico modeling have become practical and reliable tools for molecular profiling and target-based drug evaluation [24]. DFT calculations provide detailed insights into the electronic structure, chemical reactivity, and non-linear optical (NLO) properties of drug candidates [25]. Furthermore, frontier molecular orbital (HOMO–LUMO) and Molecular Electrostatic Potential (MEP) analyses offer predictive frameworks for identifying specific reactive sites [26]. When coupled with ADMET (Absorption, Distribution, Metabolism, Excretion, and Toxicity) predictions and molecular docking simulations, these theoretical approaches allow for a comprehensive assessment of drug-receptor interactions without the immediate need for extensive in vivo testing [27].

**Figure 1:**
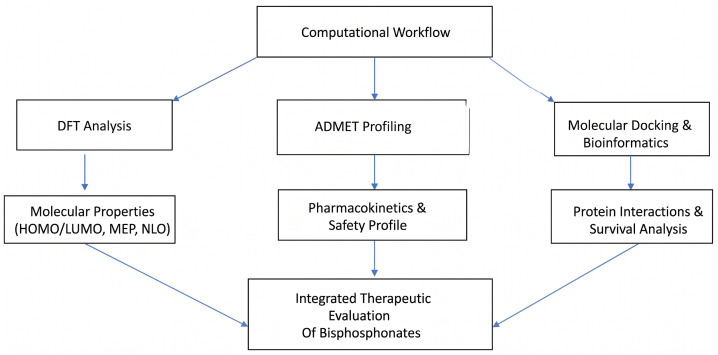
Conceptual workflow diagram integrating DFT, ADMET profiling, molecular docking, and bioinformatics to evaluate the therapeutic potential of bisphosphonate derivatives.

In light of these considerations, the present study seeks to comparatively investigate the structural, electronic, and biological binding properties of alendronate, risedronate, and zoledronate. Through the use of DFT calculations, ADMET profiling, and molecular docking analyses targeting the PDB ID: 1A52 (*ER-α*/*ESR1*) [28], 2FSZ (*ER-β*/*ESR2*) [29], 1MIU (*BRCA2*) [30], 1GKC (*MMP-9*) [31], 6QEX (*ABCB1*/*MDR*) [32], 3VHE (*VEGFR*/*KDR*) [33], 1BNL (*COL18A1*) [34], 4E4D (*OPG*/*TNFRSF11B*) [35], 3URF (*RANKL*/*TNFRSF11*) [36], 2Q80 (*GGPS1*) [37], and 2F8C (*FPPS*) [38], this research aims to provide a computational perspective on how structural variations among these specific bisphosphonates influence their chemical reactivity, pharmacokinetic profiles, and binding affinities in the context of breast cancer and osteoporosis. The present study comparatively investigated the structural, electronic, and biological binding properties of alendronate, risedronate, and zoledronate. Through the use of DFT calculations, ADMET profiling, molecular docking analyses, and bioinformatics approaches, this study aimed to provide a computational perspective on how the structural variations among these specific bisphosphonates influence their chemical reactivity, pharmacokinetic profiles, and binding affinities in the context of breast cancer and osteoporosis. To provide a clear overview of the computational strategy employed in this study, a conceptual workflow has been presented in Figure 1.

## 2. Materials and Methods

### 2.1. DFT, ADMET and Molecular Docking Analysis

The chemical structures of risedronate, zoledronate, and alendronate used in this study were retrieved from the PubChem database, https://pubchem.ncbi.nlm.nih.gov/ accessed on 14 June 2026), and their three-dimensional (3D) structures were optimized prior to computational analyses. The crystal structures of the target proteins were obtained from the RCSB Protein Data Bank (PDB), including PDB ID: 1A52 (*ER-α*/*ESR1*) [28], 2FSZ (*ER-β*/*ESR2*) [29], 1MIU (*BRCA2*) [30], 1GKC (*MMP-9*) [31], 6QEX (*ABCB1*/*MDR*) [32], 3VHE (*VEGFR*/*KDR*) [33], 1BNL (*COL18A1*) [34], 4E4D (*OPG*/*TNFRSF11B*) [35], 3URF (*RANKL*/*TNFRSF11*) [36], 2Q80 (*GGPS1*) [37], and 2F8C (*FPPS*) [38]. These targets were selected due to their roles in breast cancer progression, Osteoptoz activity, and bone-osteoporosis-related signaling pathways. Prior to docking, protein structures were prepared by removing water molecules, co-crystallized ligands, and heteroatoms, followed by the addition of polar hydrogen atoms and Kollman charges. The pharmacokinetic and toxicological properties of the compounds were evaluated using ADMETlab 2.0 [39] to assess absorption, distribution, metabolism, excretion, and toxicity (ADMET) profiles. Molecular docking studies were performed using AutoDock Vina (version 2021) [40] to predict ligand–protein binding affinities. The docking results were visualized and analyzed using BIOVIA Discovery Studio Visualizer (version 2016) [41]. For each ligand–protein complex, the optimal docking pose for 2D and 3D visualization was selected based primarily on the lowest binding energy (most negative docking score), corroborated by the presence of the most favorable and dense non-covalent interaction networks (e.g., hydrogen bonding and hydrophobic contacts) within the active site. Given the exploratory and multi-target nature of this study, evaluating the binding profiles of three specific bisphosphonates across an 11-protein network, the active site for each target was strictly defined without a separate redocking procedure. Instead, grid box dimensions and centers were meticulously established using the spatial coordinates of native co-crystallized ligands and literature-reported critical catalytic residues from the RCSB PDB structures. The generation of 33 consistent, highly detailed ligand-receptor interaction profiles across multiple pathways was considered a sufficiently robust theoretical framework when evaluated alongside the integrated DFT and bioinformatics findings. To ensure methodological reproducibility, specific AutoDock Vina search parameters were standardized. The exhaustiveness parameter was set to 8 to balance computational efficiency with comprehensive conformational sampling. To define the binding pockets across the 11 diverse target proteins, a standardized grid box dimension of approximately 30 × 30 × 30 Å was utilized. The center of each grid box was specifically positioned over the native co-crystallized ligand or the literature-defined critical catalytic residues, ensuring that the entire active site cavity was sufficiently covered to accommodate the three bisphosphonate ligands. In addition, DFT calculations were performed using the Gaussian 09 software package [42] to investigate the electronic structure and reactivity properties of the compounds. The geometries of the ligands were fully optimized, and frontier molecular orbital (HOMO–LUMO) analyses were carried out to evaluate chemical reactivity and stability. Given the highly ionized nature of bisphosphonates at physiological *pH*, ligand protonation states were carefully considered. For molecular docking studies, the ligands were prepared in their physiologically relevant polyanionic states (*pH*~7.4) to accurately simulate electrostatic interactions within the protein active sites. Conversely, for the DFT calculations, the geometries of the compounds were optimized in their neutral states (net charge = 0) to evaluate their intrinsic fundamental electronic, reactivity, and nonlinear optical (NLO) properties.

### 2.2. Gene Expression Analysis

The expression levels of *GGPS1*, *FDPS*, *TNFSF11* (*RANKL*), *TNFRSF11B* (*OPG*), *ESR1*, *ESR2*, KDR (*VEGFR2*), *ABCB1*, *MMP9*, *COL18A1*, and *BRCA2* in breast cancer were analyzed using transcriptomic datasets obtained from The Cancer Genome Atlas Breast Invasive Carcinoma (TCGA-BRCA) and Genotype-Tissue Expression (GTEx) databases. Data processing and differential expression analyses between tumor and normal breast tissues were conducted utilizing the GEPIA platform. To ensure data comparability, the raw RNA-Seq data were normalized using a log2(TPM + 1) transformation. Differential expression was assessed using a one-way ANOVA test integrated into the platform’s statistical algorithms. The thresholds for statistical significance were set at a *p*-value < 0.05 and an absolute log2 fold change (|log2FC|) cutoff of 1.0. Expression values were visualized using box plot graphs.

### 2.3. Kaplan–Meier Survival Analysis

The prognostic significance of the target genes was evaluated using Kaplan–Meier survival analysis performed through the Kaplan–Meier Plotter platform (Kaplan–Meier Plotter). Patients were stratified into high- and low-expression groups according to the median expression levels of each gene. Overall survival analyses were performed using Kaplan–Meier survival curve. Hazard ratios (HRs), 95% confidence intervals (CIs), and log-rank *p*-values were calculated to determine the association between gene expression levels and patient prognosis.

### 2.4. Protein–Protein Interaction (PPI) Network Analysis

Protein–protein interaction analysis was performed using the STRING database (STRING Database) to investigate the functional interactions among the studied genes. The interaction network was generated based on experimentally validated and predicted protein interactions. Network characteristics, including the number of nodes, number of edges, average node degree, local clustering coefficient, and PPI enrichment *p*-value, were calculated. Functional interactions among proteins were visualized using color-coded interaction lines representing different types of evidence. A PPI enrichment *p*-value < 0.05 was considered statistically significant.

### 2.5. GeneMANIA Functional Network Analysis

GeneMANIA analysis was performed using the GeneMANIA (version 3.6.0) platform (GeneMANIA) to identify functionally related genes and interaction networks associated with the target genes. The analysis evaluated multiple interaction categories, including physical interactions, co-expression, pathway associations, shared protein domains, and genetic interactions. Additional genes associated with the query genes were automatically identified using the GeneMANIA (version 3.6.0) algorithm. Interaction networks were visualized graphically, and the relative contribution of each interaction type was calculated and presented as a percentage.

### 2.6. Integrated Bioinformatics Evaluation

All bioinformatics findings were evaluated to investigate the potential roles of the studied genes in breast cancer progression. Particular attention was paid to pathways associated with mevalonate metabolism, hormone receptor signaling, angiogenesis, extracellular matrix remodeling, and the *RANK*/*RANKL*/*OPG* signaling axis. The combined analyses aimed to determine the potential prognostic and biological significance of the genes investigated in breast cancer.

## 3. Results and Discussion

**Figure 2:**
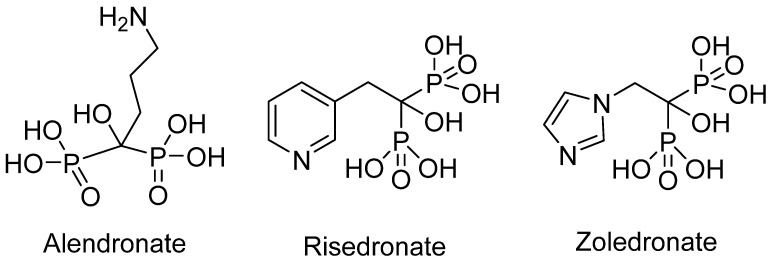
Chemical structures of alendronate, risedronate, and zoledronate.

As shown in Figure 2, alendronate is characterized by a flexible aliphatic side chain, whereas risedronate contains a heteroaromatic pyridine ring, and zoledronate incorporates an imidazole ring with higher electron density. These distinct structural features significantly influence the electronic distribution, polarity, and coordination behavior of each compound. Consequently, the observed variations in molecular architecture are directly reflected in their electronic properties and docking interactions, providing a coherent basis for interpreting the differences in binding affinity and reactivity among the studied bisphosphonates.

### 3.1. HOMO–LUMO Analysis

The HOMO–LUMO approach is evaluated within the framework of Frontier Molecular Orbital (FMO) theory, which is employed to elucidate the electronic structure of molecules [43]. According to this theory, the HOMO (Highest Occupied Molecular Orbital) represents the electron-donating tendency, whereas the LUMO (Lowest Unoccupied Molecular Orbital) indicates the electron-accepting capacity [44]. The energy gap (ΔE) between the HOMO and LUMO is considered a crucial criterion for assessing the chemical stability and reactivity of a molecule. Theoretically, a narrow HOMO–LUMO energy gap (ΔE) implies that electron transfer occurs more readily, thereby indicating that the molecule exhibits higher chemical reactivity and lower kinetic stability. Conversely, large ΔE values are associated with enhanced electronic stability and diminished reactivity [45].

Table 1 presents the frontier molecular orbital (FMO) energies (E_HOMO_ and E_LUMO_) of alendronate, risedronate, and zoledronate, calculated at the B3LYP level, along with the derived global reactivity descriptors. These quantum chemical parameters provide comprehensive insights into the electronic structures, chemical stabilities, and reactivity tendencies of the investigated molecules. As shown in Table 1, the HOMO–LUMO energy gap (ΔE) exhibited significant variations among the compounds. Risedronate exhibited the highest ΔE value (6.7468 eV), indicating the highest kinetic stability and lowest chemical reactivity within the series. In contrast, zoledronate displayed the lowest ΔE value (2.9669 eV), suggesting higher electronic softness and increased chemical reactivity. Alendronate, with a ΔE value of 5.1627 eV, lies between these two extremes and exhibits moderate stability and reactivity.

Consistent with these findings, the ionization potential (I) and electron affinity (*A*) values further support the electronic behaviors of the compounds. Risedronate exhibited the highest ionization potential (I = 6.8809 eV), indicating a greater resistance to electron removal. In contrast, zoledronate exhibited the highest electron affinity (*A* = 2.9102 eV), indicating a strong tendency to accept electrons. Alendronate exhibited an intermediate electronic profile for both parameters. Similarly, the chemical hardness (*η*) and softness (s) values corroborate these trends; risedronate, with the highest hardness value (*η* = 3.3734 eV), emerges as the most stable and least reactive compound, whereas zoledronate, with the highest softness value (*s* = 0.3370), exhibits a more reactive and highly polarizable character. Additionally, the electrophilicity index (ω) indicates that alendronate (ω = 22.8308) possesses the strongest electrophilic character among the studied compounds.

**Figure 3:**
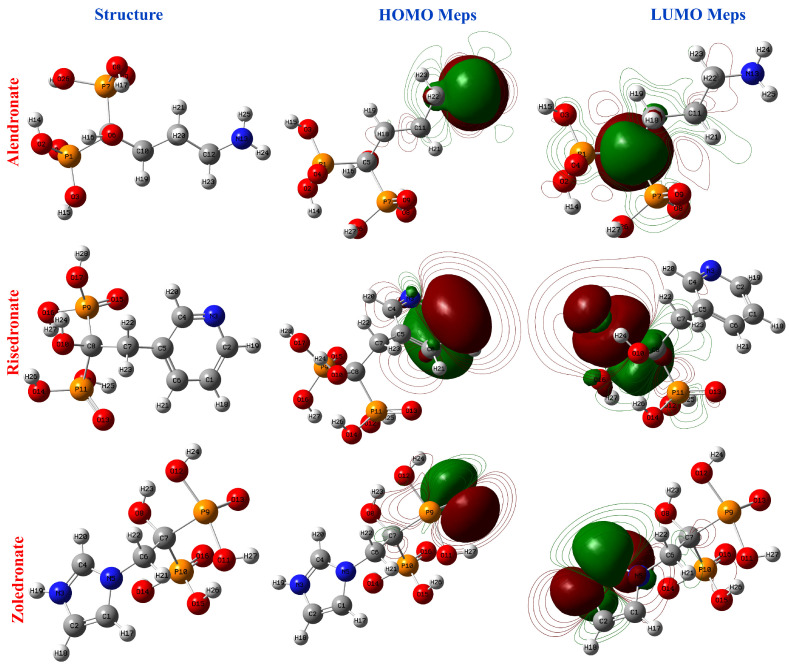
Optimized 3D geometries and HOMO and LUMO surface maps of the investigated compounds obtained at the DFT/B3LYP/6-31G(d,p) theoretical level.

Figure 3 illustrates the optimized three-dimensional molecular structures and HOMO–LUMO surface distributions obtained at the B3LYP/6-31G(d,p) level. The HOMO electron density is predominantly localized on the oxygen- and nitrogen-containing functional groups, indicating their role as electron-donating sites. In contrast, the LUMO distribution is mainly concentrated along the molecular osteoporosis and particularly on the phosphonate groups, suggesting that these regions act as potential sites for electrophilic attacks. Zoledronate exhibited a more localized orbital distribution and a higher degree of polarization, whereas risedronate showed a more delocalized electronic structure. Alendronate exhibited intermediate behavior between these two extremes. These visual results are consistent with the global reactivity descriptors reported in Table 1, further supporting the structure–reactivity relationship of the molecules.

Overall, the results clearly demonstrate that risedronate is the most electronically stable compound, zoledronate is the most reactive, and alendronate exhibits intermediate characteristics. These findings are in good agreement with the calculated global reactivity descriptors and provide important insights into the structure–reactivity relationships of the investigated compounds, which may also be relevant to their potential biological activity profiles.

### 3.2. Molecular Electrostatic Potential (MEP) Analysis

The Molecular Electrostatic Potential (MEP) is one of the fundamental conceptual tools in quantum chemical calculations, widely employed to elucidate the electronic structure, chemical reactivity tendencies, and specific interaction sites of molecules. MEP surfaces provide a topological representation of the charge distribution in three-dimensional space, enabling a qualitative identification of electrophilic and nucleophilic reactive centers within a molecule [46]. When visualized using a standard color scale, regions characterized by high electron density and susceptibility to electrophilic attack are depicted by negative potential values (red), whereas electron-deficient regions prone to nucleophilic attack are represented by positive potential values (blue). In particular, in rational drug design and computational pharmacology studies, MEP analysis plays a crucial role in understanding molecular recognition processes, evaluating electrostatic complementarity between ligands and target receptors, predicting potential hydrogen-bonding sites, and characterizing weak non-covalent intermolecular interactions [47]. In this context, MEP surfaces also provide a theoretical framework for rationalizing molecular docking results by offering detailed insights into the electrostatic interaction patterns governing ligand–receptor binding affinity and specificity [48].

**Figure 4:**
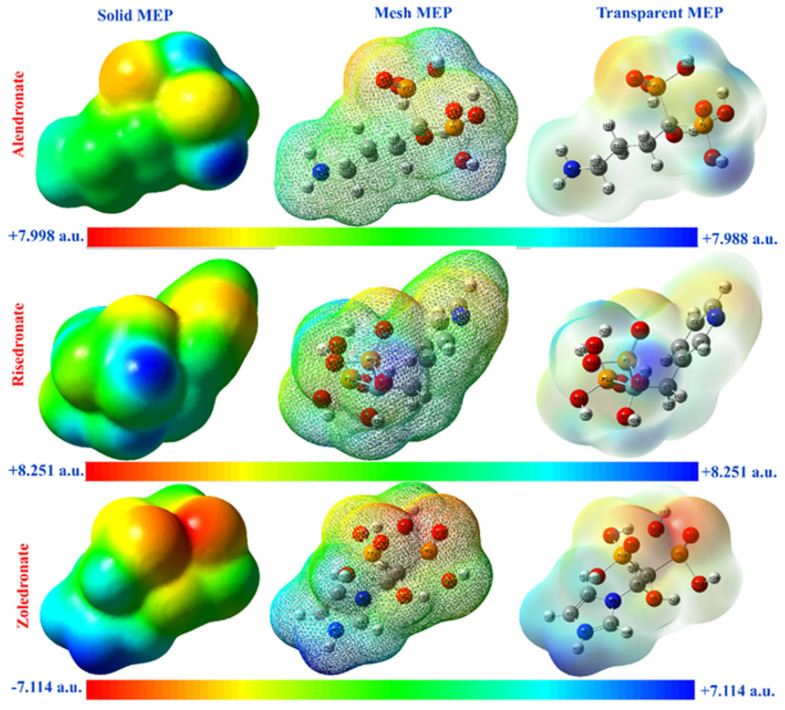
Molecular electrostatic potential (MEP) surfaces of the investigated compounds calculated at the DFT/B3LYP/6-31G(d,p) level. Different visualization modes (solid, mesh, and transparent) illustrate the charge distribution and potential binding regions around the molecules.

The Molecular Electrostatic Potential (MEP) surfaces of the investigated compounds, calculated at the DFT/B3LYP/6-31G(d,p) level of theory, are presented in Figure 4 to evaluate their electronic reactivity centers and receptor-binding potentials. The surfaces obtained using different visualization modes (solid, mesh, and transparent representations) employ a color scale in which electron-rich regions favorable for electrophilic attack are depicted in red (negative potential) and electron-deficient regions prone to nucleophilic attack are represented in blue (positive potential). From a quantitative standpoint, the numerical values at the extrema of the color scale in Figure 4 (expressed in atomic units, a.u.) correspond to the maximum (negative extreme) and minimum (positive extreme) electron densities on the molecular surfaces. Analysis of the electrostatic potential ranges reveals that risedronate (−8.251 a.u. to +8.251 a.u.) exhibits the widest potential difference, followed by alendronate (−7.998 a.u. to +7.988 a.u.) and zoledronate (−7.114 a.u. to +7.114 a.u.).

The pronounced negative and positive extrema observed for risedronate indicate more extensive surface polarization and charge separation than those of the other compounds. From both thermodynamic and electronic perspectives, this enhanced polarization suggests that risedronate can establish stronger electrostatic interactions and more stable hydrogen bonds, acting as both hydrogen bond donors and acceptors, within the binding cavities of target proteins. Accordingly, these electrostatic potential differences provide a theoretical basis for rationalizing the variations in the binding affinity observed in molecular docking studies in terms of the intrinsic electronic properties of the molecules. In all three bisphosphonate derivatives (alendronate, risedronate, and zoledronate), the most electron-rich regions were predominantly localized on the bisphosphonate groups and oxygen atoms of the hydroxyl functionalities attached to the central carbon atom. These extended red regions highlight the strong hydrogen-bond-acceptor capacity of the molecules within the active sites of the target proteins. In contrast, the most electron-deficient (blue) regions are mainly localized on the hydrogen atoms associated with the primary amine group in alendronate, the pyridine ring in risedronate, and the imidazole ring in zoledronate. This specific charge distribution pattern underlies the formation of conventional hydrogen bonds and electrostatic (van der Waals) interactions with polar residues in protein active sites, thereby providing direct support for the binding affinity results obtained from the molecular docking analyses.

### 3.3. Non-Linear Optical Properties (NLO)

Nonlinear optical (NLO) phenomena refer to physical processes in which the polarization response of a material becomes nonlinear with respect to an applied electric field under the influence of a strong electromagnetic field. This behavior gives rise to important optical effects such as second-harmonic generation (SHG), frequency mixing, and intensity-dependent refractive index changes [49]. In general, NLO behavior is described by a power series expansion of the form *P* = *αE* + *βE*^2^ + *γE*^3^, where α represents the linear polarizability, while *β* and *γ* denote the first- and second-order hyperpolarizabilities, respectively, which are the key parameters governing NLO activity [50]. In recent years, materials exhibiting NLO properties have attracted considerable attention due to their potential applications in photonics, optoelectronics, optical switching, data storage, and frequency conversion, particularly SHG [51]. In this context, organic and organometallic compounds have been extensively investigated owing to their tunable NLO responses enabled by molecular design strategies such as donor–π–acceptor (D–π–A) architectures, extended conjugation, and intramolecular charge transfer (ICT) [52].

In the present study, the nonlinear optical (NLO) properties of alendronate, risedronate, and zoledronate were theoretically investigated using the B3LYP method. Detailed computational results, including dipole moment components (*μx*, *μy*, *μz*), polarizability tensor elements (*α_xx_*, *α_yy_*, *α_zz_*, etc.), and hyperpolarizability components (*β_xxx_*, *β_xxy_*, *β_xyz_*, etc.), are provided in Tables S1–S3 in the Supporting Information. The calculated dipole moment (*μ*), mean polarizability (*α*), and first-order hyperpolarizability (*β*) values were analyzed to comparatively evaluate the NLO behavior of the studied molecules (Table 2). The results show that the dipole moment values followed the order zoledronate (8.7051 D) > alendronate (6.1039 D) > risedronate (3.5672 D), indicating that zoledronate possessed the highest molecular polarity and the most pronounced charge separation, and was therefore expected to exhibit the strongest response to external electric fields. Risedronate, which has the lowest dipole moment, displays a comparatively weaker polar character. Regarding the mean polarizability (*α*), risedronate (−107.3774 au) exhibited the highest absolute value, followed by alendronate (−97.0397 au) and zoledronate (−96.7631 au), indicating that risedronate has a more easily deformable electron cloud and higher electronic flexibility under an external electric field.

When the first-order hyperpolarizability (*β*), the most critical parameter for NLO performance, is considered, zoledronate (1.20 × 10^−30^ esu) exhibits the highest value, indicating the strongest nonlinear optical response among the investigated compounds. Alendronate (6.18 × 10^−30^ esu) showed moderate NLO activity, whereas risedronate (3.57 × 10^−30^ esu) presented the lowest hyperpolarizability. Table 2 also compares the calculated NLO parameters of the investigated compounds with those of urea, which is commonly used as a standard reference because of its well-established NLO properties. The dipole moment values of all the studied molecules were significantly higher than that of urea (1.3197 D), indicating substantially enhanced molecular polarity and charge separation. Among them, zoledronate exhibited the highest dipole moment, followed by alendronate and risedronate, all of which exceeded the reference value. A more pronounced difference was observed for the first-order hyperpolarizability (*β*), which is the most critical parameter for NLO performance. The *β* value of urea (0.1947 × 10^−30^ esu) is several orders of magnitude lower than those of the investigated compounds. In particular, alendronate (6.18 × 10^−30^ esu), risedronate (3.57 × 10^−30^ esu), and zoledronate (1.20 × 10^−30^ esu) exhibited significantly enhanced hyperpolarizability values compared with urea, confirming their superior nonlinear optical responses.

Overall, the obtained results demonstrate that alendronate, risedronate, and zoledronate not only exhibit electronically active structures but also possess significantly enhanced nonlinear optical (NLO) properties compared to urea. These findings suggest that the investigated compounds may serve as potential candidates for advanced nonlinear optical and photonic applications. A comparative evaluation indicated that zoledronate exhibited the strongest NLO character in terms of dipole moment and hyperpolarizability, whereas risedronate showed the weakest nonlinear optical response. In contrast, an inverse trend was observed for polarizability, where risedronate demonstrated the highest electronic deformability under an external electric field. These results indicate that the structural differences among the investigated bisphosphonate derivatives have a pronounced and multifaceted influence on their nonlinear optical properties.

### 3.4. ADMET Analysis

ADMET (absorption, distribution, metabolism, excretion, and toxicity) analyses conducted to evaluate the bioavailability, pharmacokinetic behavior, and safety profiles of drug candidates are considered an important computational approach for predicting the therapeutic efficacy potential of molecules [53]. In particular, oral bioavailability, membrane permeability, metabolic stability, and toxicity parameters are regarded as critical indicators of the clinical applicability of a compound [54].

In this study, the physicochemical and ADMET properties of alendronate, risedronate, and zoledronate were comparatively assessed (Table 3). As shown in Table 3, all the investigated compounds exhibited relatively low molecular weights (249.02–282.01 g/mol). According to previous studies, low molecular weight generally provides advantages in terms of absorption and biological accessibility of drugs. However, the relatively high number of hydrogen bond acceptors (nHA) and donors (nHD), along with elevated topological polar surface area (TPSA > 130 Å^2^) values, suggest that these molecules possess highly polar characteristics. High TPSA values restrict passive diffusion across biological membranes and may reduce oral permeability. The evaluation of lipophilicity parameters demonstrated that all compounds possessed negative logP values. Notably, the markedly low logP value of alendronate (−3.082) reflects its hydrophilicity. It has been reported that negative or low logP values, although favorable for aqueous solubility, may reduce cellular membrane permeability. This characteristic may be one of the key factors underlying the limited intestinal absorption of bisphosphonates. Although the human intestinal absorption (HIA) values were predicted to be high (1.0) for all compounds, the relatively low Caco-2 permeability values, particularly for risedronate and zoledronate, are noteworthy. The Caco-2 model is widely used as an in vitro predictor of intestinal epithelial permeability, and low permeability values indicate limited capacity for passive membrane diffusion. This observation is consistent with the low oral bioavailability of bisphosphonates.

Regarding distribution parameters, the low blood–brain barrier (BBB) permeability values suggest that these molecules exhibit limited penetration into the central nervous system. Given their bone-targeted therapeutic effects, this feature may be advantageous. Moreover, low-to-moderate plasma protein binding (PPB) values indicate that a relatively higher free drug fraction may remain available in the systemic circulation. From a metabolic perspective, the low predicted inhibition probabilities for CYP2D6 and CYP3A4 suggest limited interaction with the cytochrome P450 enzyme system, indicating a potentially low risk of drug–drug interactions. Furthermore, the predicted short half-life (T_1/2_) values suggest that these compounds may be eliminated relatively rapidly from the systemic circulation. However, owing to the strong affinity of bisphosphonates for the bone matrix, their biological effects have been reported to persist for extended periods. Toxicity assessment revealed a low probability of hERG channel blockade, suggesting a minimal risk of cardiotoxicity. Similarly, low AMES mutagenicity and hepatotoxicity predictions indicate a favorable safety profile. The predicted rat oral acute toxicity values were also relatively low, further supporting the low acute toxicity potential of these compounds.

Overall, the ADMET results presented in Table 3 indicate that alendronate, risedronate, and zoledronate have acceptable pharmacokinetic and safety profiles. Nevertheless, their high polarity and low lipophilicity appear to limit membrane permeability, representing a major restricting factor that may adversely affect the oral bioavailability. At the same time, these characteristics may contribute to their selective accumulation in bone tissue and support their potential applicability in therapeutic strategies related to bone-targeted disorders and osteoporosis.

**Figure 5:**
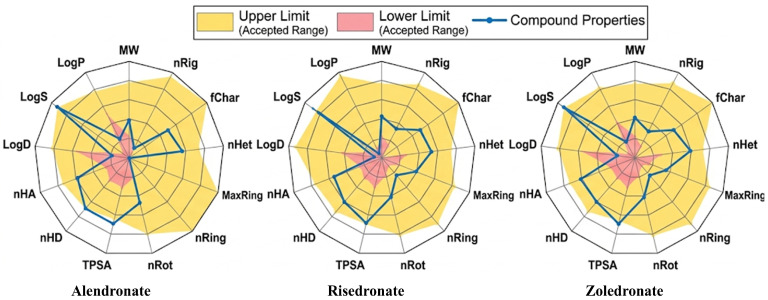
Color regions and physicochemical parameters of the Alendronate, Risedronate and Zoledronate compounds.

Figure 5 compares the physicochemical properties and drug-like parameters of alendronate, risedronate, and zoledronate using radar plots. In the radar diagrams, the blue region represents the calculated molecular properties of the compounds, whereas the yellow and pink regions correspond to the recommended upper and lower threshold ranges, respectively. Overall, the three bisphosphonate derivatives exhibited similar physicochemical profiles, although notable differences were observed for certain parameters. The radar plot of alendronate indicates that its molecular weight (MW) and topological polar surface area (TPSA) values are within acceptable ranges. However, the considerably low logP and logD values reflect the highly hydrophilic nature of these compounds. Previous studies have suggested that low logP values may improve aqueous solubility while simultaneously reducing passive diffusion across the biological membranes. Furthermore, the relatively high number of hydrogen bond donors (nHD) and acceptors (nHA) suggests a strong capacity for polar interactions. This characteristic is expected for bisphosphonates, which are known to exhibit a high affinity for the bone mineral matrix.

The radar profile of risedronate revealed a comparatively more balanced lipophilicity profile than that of the other compounds. In particular, its relatively higher logP value compared with that of alendronate may confer a potential advantage in terms of membrane permeability. Nevertheless, the persistently high TPSA and hydrogen bonding parameters indicate that the molecule retains its pronounced polar character. Moreover, the parameters associated with molecular rigidity and ring structure remained within the recommended ranges, suggesting that risedronate may possess a balanced structural stability profile. The physicochemical profile of zoledronate demonstrated that, despite its high polarity, most molecular descriptors remained within the recommended limits. In particular, the elevated TPSA and hydrogen-bonding capacity further support the hydrophilic nature of the molecule. Additionally, the relatively favorable logS (solubility) parameter suggests a good aqueous solubility. The nitrogen-containing heterocyclic structure of zoledronate (imidazole ring) may also represent an important structural advantage that enhances interactions with biological targets.

Overall, Figure 5 demonstrates that the three bisphosphonate derivatives possess acceptable physicochemical characteristics in terms of drug-like properties. However, their high polarity and low lipophilicity may limit membrane permeability, which is consistent with the low oral bioavailability of bisphosphonates reported in the literature. Nevertheless, the presence of highly hydrophilic structures and polar functional groups may promote strong binding to hydroxyapatite surfaces in bone tissue, thereby providing a therapeutic advantage, particularly in the treatment of osteoporosis and osteoclast-associated breast cancer.

### 3.5. Molecular Docking Analysis

Molecular docking analysis is one of the most widely used computational approaches for evaluating the binding tendency and interaction strength of small molecules within the active sites of target proteins [55]. This method contributes to the prediction of potential biological activity by calculating the binding energy of ligand–protein complexes. According to the literature, more negative docking scores generally indicate stronger and more stable binding of the ligand to the target protein [56].

In the present study, the binding affinities of alendronate, risedronate, and zoledronate against eleven protein targets associated with Osteoptoz and breast cancer progression, including the PDB ID: 1A52 (*ER-α*/*ESR1*) [28], 2FSZ (*ER-β*/*ESR2*) [29], 1MIU (*BRCA2*) [30], 1GKC (*MMP-9*) [31], 6QEX (*ABCB1*/*MDR*) [32], 3VHE (*VEGFR*/*KDR*) [33], 1BNL (*COL18A1*) [34], 4E4D (*OPG*/*TNFRSF11B*) [35], 3URF (*RANKL*/*TNFRSF11*) [36], 2Q80 (*GGPS1*) [37], and 2F8C (*FPPS*) [38], were investigated using molecular docking analysis (Table 4). In general, the docking scores ranged from −5.30 to −7.90 kcal/mol, indicating favorable ligand–protein interactions across all investigated targets. Among the evaluated bisphosphonates, zoledronate exhibited the strongest overall binding profile, yielding the most favorable docking scores against *FPPS* (PDB ID: 2F8C, −7.90 kcal/mol), *ER-β* (PDB ID: 2FSZ, −7.80 kcal/mol), and *COL18A1* (PDB ID: 1BNL, −7.70 kcal/mol). The pronounced affinity of zoledronate toward *FPPS* and *GGPS1* (PDB ID: 2Q80, −7.50 kcal/mol) is particularly noteworthy, considering that nitrogen-containing bisphosphonates exert antiresorptive effects by inhibiting enzymes within the mevalonate pathway, thereby suppressing osteoclast-mediated bone resorption.

Risedronate also demonstrated considerable binding potential, particularly against *BRCA2* (PDB ID: 1MIU, −7.70 kcal/mol), *MMP-9* (PDB ID: 1GKC, −7.70 kcal/mol), *VEGFR* (PDB ID: 3VHE, −7.70 kcal/mol), and *ABCB1*/*MDR* (PDB ID: 6QEX, −7.50 kcal/mol) proteins. These findings suggest the potential involvement of risedronate in modulating pathways associated with angiogenesis, extracellular matrix remodeling, and multidrug resistance, all of which are implicated in breast cancer progression. Additionally, the favorable interactions observed with *RANKL* (PDB ID: 3URF) and *OPG* (PDB ID: 4E4D) suggest a possible contribution to osteoporosis regulation.

In contrast, alendronate generally displayed comparatively lower docking scores than risedronate and zoledronate, although it maintained favorable interactions with *VEGFR* (PDB ID: 3VHE, −7.40 kcal/mol), *MMP-9* (PDB ID: 1GKC, −7.40 kcal/mol), and *FPPS* (PDB ID: 2F8C, −7.20 kcal/mol). The relatively consistent binding affinities observed for all three compounds against *RANKL*, *OPG*, *GGPS1*, and *FPPS* further support their potential role in targeting osteoporotic mechanisms by modulating osteoclast activity and bone turnover. Collectively, the docking findings suggest that zoledronate, followed by risedronate, may possess superior binding potential toward osteoporosis-related and breast cancer-associated molecular targets, warranting further experimental validation through in vitro and in vivo studies.

**Figure 6:**
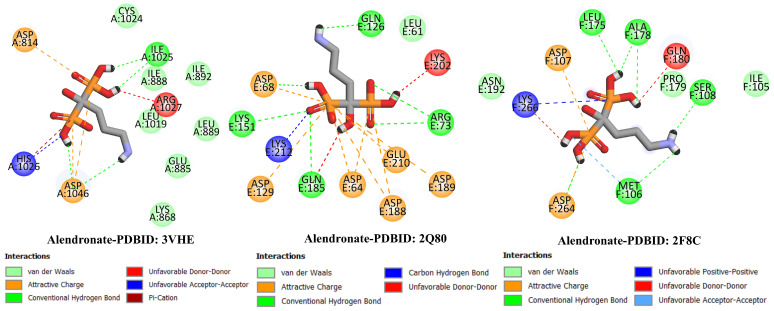
Two-dimensional (2D) interaction diagrams of alendronate with the representative protein targets showing the most significant interaction patterns.

Figure 6 illustrates the representative two-dimensional (2D) interaction profiles of alendronate with the protein targets exhibiting the most favorable binding interactions (PDB IDs: 3VHE, 2Q80, and 2F8C), while the interaction diagrams of the remaining protein targets are provided in the Supplementary Materials (Figure S1) to improve the clarity and visual quality of the manuscript. Interaction analysis demonstrated that alendronate established multiple stabilizing interactions within the active binding pockets of all selected proteins, predominantly through hydrogen bonding, electrostatic interactions, and van der Waals contact. Owing to the phosphonate functional groups present in the alendronate structure, the ligand exhibits a strong tendency to form conventional hydrogen bonds and electrostatic interactions with polar and charged amino acid residues, thereby contributing to the stabilization of the ligand–protein complexes.

In the estrogen receptor targets, *ER-α* (PDB ID: 1A52) and *ER-β* (PDB ID: 2FSZ), alendronate exhibited several hydrogen bond interactions with the surrounding amino acid residues, accompanied by van der Waals interactions that may support ligand accommodation within the receptor binding pocket. Notably, the relatively favorable docking score observed for *ER-β* suggests a comparatively stronger binding affinity than that of ER-α. Similarly, interactions observed with *BRCA2* (PDB ID: 1MIU), *MMP-9* (PDB ID: 1GKC), and *VEGFR* (PDB ID: 3VHE) indicate the establishment of stabilizing contacts within the functionally relevant regions of these proteins, potentially supporting the modulation of pathways involved in cellular proliferation, extracellular matrix remodeling, and angiogenesis.

Furthermore, alendronate displayed characteristic interactions with proteins involved in bone remodeling, including *OPG* (PDB ID: 4E4D), *RANKL* (PDB ID: 3URF), *GGPS1* (PDB ID: 2Q80), and *FPPS* (PDB ID: 2F8C). Particularly in *GGPS1* and *FPPS*, the interaction profiles revealed multiple hydrogen bonds and electrostatic contacts, which may explain the relatively favorable docking scores obtained for these enzymes. Given the established role of nitrogen-containing bisphosphonates in inhibiting mevalonate pathway enzymes, these findings further support the molecular basis of alendronate-mediated suppression of osteoclast activity and bone resorption. Overall, the interaction profiles suggest that hydrogen bonding and electrostatic interactions mediated by phosphonate groups are the principal contributors to the binding stability of alendronate across the investigated targets.

Intermolecular interaction analysis of alendronate with the selected target proteins, PDB: 2F8C (*FPPS*), PDB: 2Q80 (*GGPS1*), and PDB: 3VHE (*VEGFR*/*KDR*), demonstrated distinct binding patterns governed by hydrogen bonding, electrostatic interactions, and hydrophobic contacts (Table 5). These interaction profiles provide important insights into the molecular basis of alendronate stabilization within the active regions of proteins associated with osteoporosis and breast cancer progression.

For the PDB: 2F8C (*FPPS*) complex, alendronate stabilized through multiple conventional hydrogen bonds involving LEU-175, MET-106, SER-108, and ALA-178, with bond distances ranging from 3.24 to 5.33 Å. In addition, van der Waals interactions with PRO-179 and ILE-105 may contribute to the stabilization of the ligand in the binding pocket. Electrostatic attractive charge interactions with ASP-107 and ASP-264 further supported ligand accommodation, indicating a favorable charge complementarity. However, unfavorable donor–donor interactions with LYS-266 and GLN-180, together with unfavorable acceptor–acceptor interactions involving MET-106 and positive–positive interactions with LYS-266, suggest the presence of steric or electrostatic repulsion that may partially reduce the binding efficiency despite the favorable docking score.

In the PDB: 2Q80 (GGPS1) complex, alendronate demonstrated an extensive interaction profile characterized by several van der Waals contacts involving LYS-202, GLN-185, THR-152, LEU-155, HIS-57, GLN-126, and LEU-61, suggesting efficient ligand accommodation within the active pocket. Conventional hydrogen bonds formed with LYS-151, ARG-73, ASP-68, and ASP-64, with bond lengths ranging from 4.75 to 7.20 Å, indicating moderate intermolecular stabilization. Furthermore, attractive charge interactions with ASP-68, ASP-129, ASP-188, and ASP-64 suggest a strong contribution of electrostatic interactions, likely associated with the alendronate phosphonate groups. The additional carbon–hydrogen bond and π–alkyl interactions with LYS-151 may also contribute to complex stabilization. Nevertheless, the presence of unfavorable donor–donor interactions with LYS-212 may slightly weaken the binding conformation.

Among the investigated complexes, alendronate displayed a particularly favorable interaction profile with PDB 3VHE (*VEGFR*/*KDR*). Multiple van der Waals interactions involving CYS-1024, LEU-1019, LYS-868, ILE-888, and ILE-892 facilitated the ligand positioning within the receptor cavity. Strong conventional hydrogen bonding with HIS-1026 and ILE-1025, characterized by relatively short bond distances (3.06 and 4.13 Å, respectively), indicates stable ligand accommodation. Additionally, attractive charge interactions with ASP-814 and ASP-1046, combined with carbon–hydrogen bonding involving HIS-1026 and ASP-1046, suggest enhanced electrostatic stabilization. The presence of π–cation interactions with HIS-1026 and π–anion interactions with GLU-885 further supports favorable noncovalent interactions within the binding site. Although unfavorable donor–donor interactions with ARG-1027 and acceptor–acceptor interactions with ILE-1025 were detected, the predominance of favorable interactions suggests a stable binding mode of alendronate within the VEGFR active pocket.

Overall, the interaction analysis revealed that alendronate binding is predominantly mediated by hydrogen bonding and electrostatic interactions, particularly those involving negatively charged aspartate residues. Among the evaluated proteins, the 3VHE (*VEGFR*/*KDR*) complex exhibited the most diverse interaction profile, indicating potentially stronger ligand stabilization than the *FPPS* and *GGPS1* complexes. These findings are consistent with the docking results and further support the potential multi-target therapeutic activity of alendronate in osteoporosis and breast cancer-related pathways.

**Figure 7:**
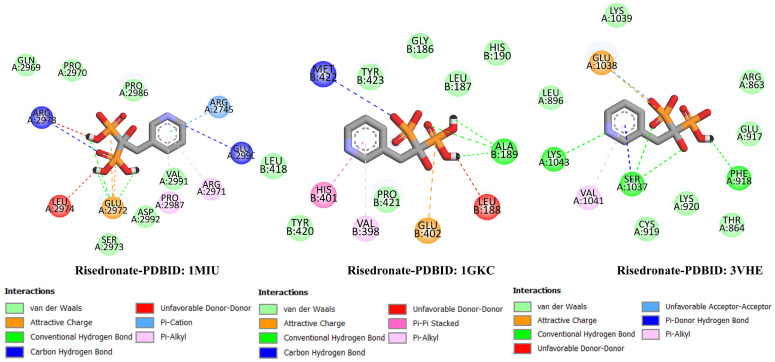
Two-dimensional (2D) interaction diagrams of risedronate with the representative protein targets showing the most significant interaction patterns.

Figure 7 shows the representative 2D ligand–receptor interaction profiles of risedronate with the protein targets exhibiting the most significant interaction patterns (PDB IDs: 1MIU, 1GKC, and 3VHE), whereas the interaction diagrams of the remaining target proteins are presented in the Supplementary Materials (Figure S2). Overall, risedronate exhibited richer and more heterogeneous interaction types than alendronate, particularly π–π stacking and hydrophobic interactions, which were significantly increased owing to its aromatic ring-containing structure. This contributes to the formation of a more stable binding conformation within the binding pocket. In the *ER-α* (1A52) and *ER-β* (2FSZ) complexes, risedronate formed π–π and π-alkyl interactions with aromatic amino acid residues, in addition to hydrogen bonds. This indicates that the ligand gains conformational stability in addition to the electrostatic fit in the steroid receptor binding pocket. The increased interactions, particularly with the hydrophobic pocket, play a significant role in increasing the binding affinity. In the *BRCA2* (1MIU) and *MMP-9* (1GKC) complexes, risedronate exhibits prominent hydrogen bond donor/acceptor properties and π-interactions via its aromatic ring, demonstrating multifaceted binding modes in the active site. This multiple interaction profile is one of the key factors that enhance protein-ligand complex stability.

In complexes formed with *ABCB1* (6QEX) and *VEGFR-2* (3VHE), risedronate showed a more balanced distribution of interactions, and hydrogen bonds and hydrophobic contacts worked together to stabilize the binding. This supports the potential pharmacological effects of the compound, particularly in relation to multiple resistance proteins and angiogenesis receptors.

In the *COL18A1* (1BNL) and *TNFRSF11B* (4E4D) complexes, risedronate formed prominent hydrogen bond networks and exhibited a conformation that fit well into the binding pocket. In particular, the strong electrostatic interactions between the phosphonate groups and polar residues enhance the binding stability. In the *RANKL* (3URF), *GGPS1* (2Q80), and *FPPS* (2F8C) complexes, risedronate provided strong stabilization through both ionic interactions and hydrophobic contacts with the aromatic ring. The dense interaction network observed, especially in the *FPPS* (2F8C) complex, is an important finding that supports the potential of risedronate to target prenylation pathways. Overall, Figure 7 shows that risedronate exhibits a strong binding profile for all target proteins through multiple interaction modes (hydrogen bonds, π–π stacking, hydrophobic, and electrostatic interactions). This multifaceted interaction suggests that risedronate is a potential candidate for modulating bone resorption and tumor progression mechanisms associated with osteoporosis.

Intermolecular interaction analysis of risedronate with the selected target proteins, namely PDB: 1MIU (*BRCA2*), PDB: 1GKC (*MMP-9*), and PDB: 3VHE (*VEGFR*/*KDR*), revealed that the ligand exhibited distinct binding profiles depending on the protein environment (Table 6). Overall, binding stability is primarily governed by hydrogen bonding, van der Waals interactions, and electrostatic forces, while additional hydrophobic and π-type interactions further contribute to complex stabilization.

In the PDB: 1MIU (*BRCA2*) complex, risedronate displayed extensive van der Waals interactions with GLN-2969, PRO-2970, PRO-2986, VAL-2991, ASP-2992, and SER-2973, indicating a broad contact surface within the protein-binding region. Conventional hydrogen bonds formed with GLU-2972 (2.76–4.90 Å) significantly contributed to ligand stabilization. In addition, attractive charge interactions with the same residue (4.45–4.65 Å) suggest a strong electrostatic complementarity. However, unfavorable donor–donor interactions involving LEU-2974 and AER-2978 may introduce steric or electrostatic constraints that partially reduce the binding efficiency. Furthermore, π–cation interactions with ARG-2745 and π–alkyl interactions with PRO-2987 and ARG-2971 contributed to additional stabilization through hydrophobic contacts.

In the PDB: 1GKC (*MMP-9*) complex, risedronate exhibited extensive van der Waals interactions with GLY-186, LEU-187, HIS-190, LEU-418, TYR-420, PRO-421, and TYR-423, indicating efficient accommodation within the active binding pocket. Conventional hydrogen bonding with ALA-189 (2.89–4.23 Å) supported strong molecular stabilization. Moreover, attractive charge interactions with GLU-402 (6.45 Å) provide an electrostatic contribution, whereas a carbon–hydrogen bond with MET-422 (4.41 Å) further enhances the complex stability. Additional π–alkyl interactions with VAL-398 and π–π stacking interactions with HIS-401 highlight the role of aromatic and hydrophobic contacts in strengthening ligand binding. Overall, this complex exhibited a balanced interaction profile dominated by hydrogen bonding and hydrophobic interactions.

In the PDB: 3VHE (*VEGFR*/*KDR*) complex, risedronate formed strong van der Waals interactions with LYS-1039, LEU-896, ARG-863, GLU-917, LYS-920, THR-864, and CYS-919, suggesting a stable position within the binding pocket. Conventional hydrogen bonds with LYS-1043, SER-1037, and PHE-918 (4.30–5.88 Å) significantly contributed to ligand stabilization. Attractive charge interaction with GLU-1038 (4.62 Å) enhanced electrostatic complementarity, while π–donor hydrogen bonding involving SER-1037 and π–alkyl interaction with VAL-1041 further supported binding stability. However, an unfavorable acceptor–acceptor interaction involving GLU-1038 may partially destabilize the binding conformation.

Overall, risedronate binding is primarily driven by hydrogen bonding and electrostatic interactions, complemented by van der Waals and π-type interactions, which enhance complex stability. Notably, the *MMP-9* (1GKC) and *VEGFR*/*KDR* (3VHE) complexes exhibited more diverse interaction networks, suggesting potentially more stable and favorable binding environments. These findings are consistent with the docking results and support the multi-target therapeutic potential of Risedronate.

**Figure 8:**
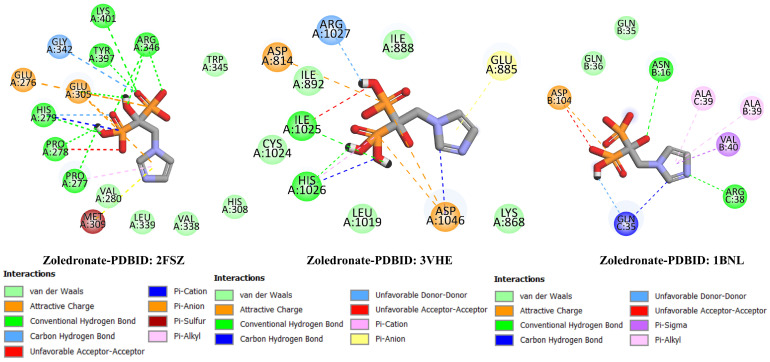
Two-dimensional (2D) interaction diagrams of zoledronate with the representative protein targets showing the most significant interaction patterns.

Figure 8 shows the representative two-dimensional (2D) interaction profiles of zoledronate with the protein targets exhibiting the most significant interaction patterns and docking performance (PDB IDs: 2FSZ, 3VHE and 1BNL), whereas the interaction diagrams of the remaining target proteins are provided in the Supplementary Materials (Figure S3). These diagrams provide a detailed representation of the hydrogen bonds, hydrophobic interactions, electrostatic interactions, and van der Waals forces formed between the ligand and amino acid residues within the binding pockets of each protein.

Overall, zoledronate demonstrated a complex and multi-interaction binding profile across all investigated protein targets. In particular, hydrogen bonding and electrostatic interactions play dominant roles in stabilizing the ligand within the active sites. The phosphonate groups of zoledronate strongly interact with polar and positively charged amino acid residues, contributing significantly to overall binding stability.

In the case of 2FSZ (*ER-β*/*ESR2*), zoledronate forms an extensive network of hydrogen bonds and electrostatic interactions, indicating tight and stable binding within the active site. This observation is consistent with the relatively high binding affinity obtained for this target (−7.80 kcal/mol), which supports the strong stabilization of the ligand–receptor complex.

Similarly, in the 3VHE (*VEGFR*/*KDR*) and 1GKC (*MMP-9*) complexes, zoledronate established both hydrogen bonding and hydrophobic contacts with key active-site residues. In addition, metalloprotein targets, such as MMP-9, may exhibit enhanced binding stability due to potential coordination interactions with metal ions. These findings suggest a relevant inhibitory potential against the proteins involved in tumor angiogenesis and metastasis.

For 1BNL (*COL18A1*) and 3URF (*RANKL*/*TNFRSF11*), zoledronate exhibited a broad interaction network involving both polar contacts and van der Waals interactions, leading to a stable ligand conformation within the binding pocket.

In contrast, interactions with 4E4D (*OPG*/*TNFRSF11B*) and 1A52 (*ER-α*/*ESR1*) were relatively fewer; however, hydrogen bonds and van der Waals forces contributed to the overall stability of these complexes.

Overall, the 2D interaction analyses (Figure 8) revealed that zoledronate exhibited multimodal binding behavior with all target proteins. The predominant role of hydrogen bonds and electrostatic interactions suggests that the phosphonate groups in the compound function as critical pharmacophores in biomolecular recognition. These results, consistent with the docking scores, support the strong binding potential of zoledronate to multiple protein targets involved in osteoporosis and breast cancer progression.

Intermolecular interaction analysis of zoledronate with the selected target proteins (PDB: 2FSZ, PDB: 1BNL, and PDB: 3VHE) revealed a complex and highly diverse binding behavior characterized by hydrogen bonding, electrostatic interactions, van der Waals forces, and multiple π-type contacts (Table 7). These interactions collectively suggest that zoledronate can effectively adapt to different protein-binding environments, contributing to its multi-target pharmacological potential.

In the PDB: 2FSZ (*ER-β*/*ESR2*) complex, zoledronate exhibited extensive van der Waals interactions with residues TRP-345, HIS-308, VAL-338, VAL-280, and LEU-339, indicating strong hydrophobic accommodation in the ligand-binding pocket. Conventional hydrogen bonds were formed with LYS-401, TYR-397, GLU-305, HIS-279, PRO-278, and ARG-346, with relatively long bond distances ranging from 3.94 to 7.41 Å, suggesting a flexible but multipoint anchoring mode. Electrostatic attractive charge interactions involving GLU A:276 and GLU A:305 further supported ligand stabilization through charge complementarity. Additional π–cation interactions with HIS-279, π–anion interactions with GLU-305, π–sulfur interactions with MET-309, and π–alkyl interactions with PRO-277 highlight a rich network of non-covalent forces contributing to the binding affinity. However, the presence of an unfavorable acceptor–acceptor interaction with PRO-278 suggests localized steric or electronic repulsion within the binding sites.

For the PDB: 1BNL (*COL18A1*) complex, zoledronate showed van der Waals interactions mainly with GLN-35 and GLN-36, indicating a relatively limited hydrophobic contact network compared to the other complexes. Conventional hydrogen bonding with ASN-16 and ARG-38 suggested specific but fewer anchoring points with moderate bond distances. A carbon–hydrogen bond with GLN-35 further contributes to stabilization. However, the presence of unfavorable donor–donor interactions with GLN-35 and unfavorable acceptor–acceptor interactions with ASP-104 indicates partial instability in certain regions of the binding conformation. A π–sigma interaction observed with VAL B:40 provides additional, albeit weaker, stabilization.

In the PDB: 3VHE (*VEGFR*/*KDR*) complex, zoledronate exhibited a strong and well-organized interaction network. Van der Waals interactions with ILE-888, ILE-892, CYS-1024, LEU-1019, and LYS-868 suggest effective positioning within the active-binding pocket. Conventional hydrogen bonds with ILE-1025 and HIS-1026, characterized by relatively short bond distances (3.71–4.13 Å), indicate strong ligand anchoring in the active site. Attractive charge interactions with ASP-814 and ASP-1046 further enhanced the electrostatic complementarity and binding stability. Additional carbon–hydrogen bonds involving HIS-1026 and ASP-1046, along with π–cation interactions with HIS-1026 and π–anion interactions with GLU-885, further reinforced the ligand–protein complex. However, unfavorable donor–donor interactions with ARG-1027 and unfavorable acceptor–acceptor interactions with ILE-1025 may introduce minor destabilizing effects.

Overall, zoledronate exhibited a highly versatile binding profile across all three target proteins, with hydrogen bonding and electrostatic interactions serving as the primary stabilizing forces, complemented by van der Waals and π-type interactions. Among the studied targets, the PDB: 3VHE (*VEGFR*/*KDR*) complex exhibited the most balanced and dense interaction network, suggesting the most stable binding environment for zoledronate. These findings are consistent with the docking results and further support the multi-target therapeutic potential of zoledronate in osteoporosis and breast cancer-related pathways.

### 3.6. Bioinformatics Analysis

**Figure 9:**
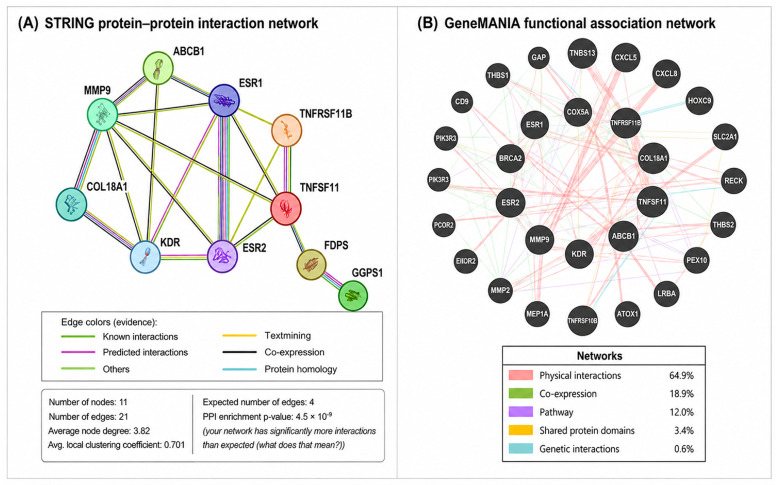
STRING and GeneMANIA network analyses of bisphosphonate-related breast cancer genes. (**A**) STRING protein-protein interaction network showing significant functional connectivity among *ESR1*, *ESR2*, *KDR*, *MMP9*, *COL18A1*, *TNFSF11*, *TNFRSF11B*, *FDPS*, *GGPS1*, and *ABCB1* (*p* = 4.5 × 10^−9^). (**B**) GeneMANIA network illustrating physical interactions, co-expression, pathway associations, shared protein domains, and genetic interactions among the analyzed genes and their related partners. These networks highlight the involvement of the mevalonate pathway, hormone receptor signaling, angiogenesis, extracellular matrix remodeling, and *RANK*/*RANKL*/*OPG* signaling in breast cancer.

STRING and GeneMANIA network analyses revealed strong functional and physical interactions among the investigated genes. In the STRING protein–protein interaction (PPI) analysis, 11 nodes and 21 interactions were identified. Although the expected number of interactions in the network was four, the observed number was substantially higher, indicating that the analyzed genes are biologically interconnected in a meaningful manner (PPI enrichment *p* = 4.5 × 10^−9^). The average node degree and local clustering coefficient of the network were 3.82 and 0.701, respectively. Network analysis identified *ESR1*, *ESR2*, *KDR*, *MMP9*, *COL18A1*, *TNFSF11*, and *TNFRSF11B* as central hub genes. In particular, the multiple connections exhibited by ESR1 and ESR2 suggest that hormone receptor–associated signaling mechanisms may play an important role in network organization (Figure 9A).

GeneMANIA analysis demonstrated that gene–gene relationships were predominantly composed of physical interactions (64.93%), followed by co-expression (18.96%), pathway associations (12.02%), shared protein domains (3.49%), and genetic interactions (0.60%). Additional genes related to the network included *VEGFC*, *THBS2*, *CXCL5*, *CXCL6*, *TNFSF13*, *BRCA2*, and *HOXC8*. These findings suggest that the analyzed genes may collectively participate in biological processes related to tumor progression, cell proliferation, angiogenesis, extracellular matrix remodeling, and hormone-related signaling pathways (Figure 9B).

**Figure 10:**
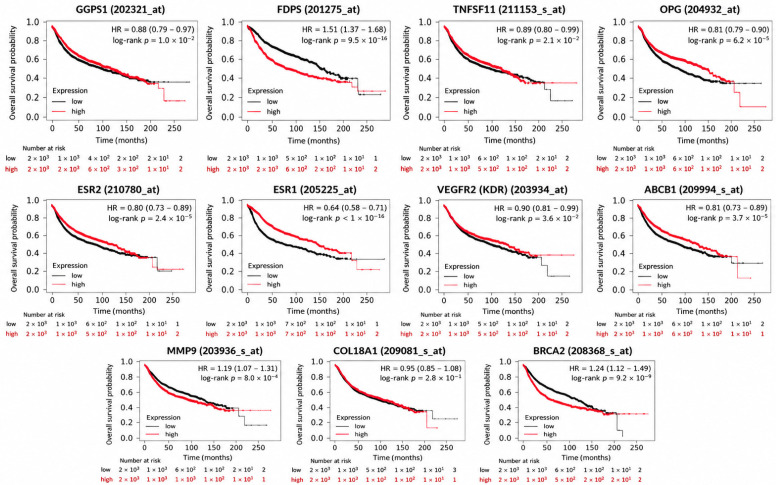
Survival and expression analyses of candidate breast cancer genes. Kaplan–Meier survival curves demonstrating the prognostic impact of GGPS1, FDPS, TNFSF11, *TNFRSF11B*, *ESR1*, *ESR2*, *KDR*, *ABCB1*, *MMP9*, *COL18A1*, and *BRCA2* expression in breast cancer patients.

**Figure 11:**
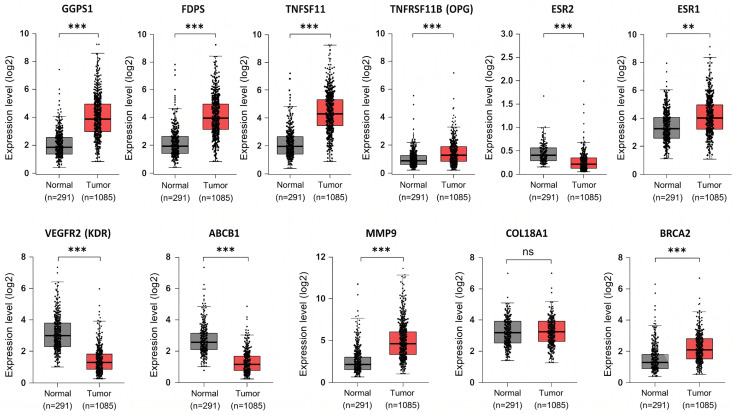
Survival and expression analyses of candidate breast cancer genes. Comparative gene expression analysis between breast cancer and normal breast tissues showing significant upregulation of *GGPS1*, *FDPS*, *TNFSF11*, *ESR1*, *MMP9*, and *BRCA2*, and significant downregulation of *ESR2*, *KDR*, and *ABCB1* in tumor tissues. *COL18A1* expression did not differ significantly between groups. Statistical significance is denoted as follows: ns, not significant (*p* ≥ 0.05); **, *p* < 0.01; ***, *p* < 0.001. Individual dots represent single patient samples.

Kaplan–Meier survival analyses revealed that the investigated genes were significantly associated with breast cancer prognosis (Figure 10). High FDPS expression was associated with poor survival (HR = 1.51, *p* = 9.5 × 10^−16^). Similarly, elevated expression levels of *MMP9* (HR = 1.19, *p* = 0.00089) and *BRCA2* (HR = 1.34, *p* = 9.2 × 10^−9^) were associated with worse overall survival. In contrast, higher expression levels of *GGPS1* (HR = 0.88, *p* = 0.01), *TNFSF11*/*RANKL* (HR = 0.89, *p* = 0.021), *TNFRSF11B*/*OPG* (HR = 0.81, *p* = 6.2 × 10^−5^), *ESR2* (HR = 0.80, *p* = 2.4 × 10^−5^), *ESR1* (HR = 0.64, *p* < 1 × 10^−16^), *KDR*/*VEGFR2* (HR = 0.90, *p* = 0.036), and ABCB1 (HR = 0.81, *p* = 3.7 × 10^−5^) were associated with improved survival. No significant association was observed between *COL18A1* expression and survival (HR = 0.95, *p* = 0.28) (Figure 10). Gene expression analysis revealed that *GGPS1*, *FDPS*, *TNFSF11* (*RANKL*), *ESR1*, *MMP9*, and *BRCA2* were significantly upregulated in breast cancer tissues compared to normal tissues (Figure 11). In contrast, the expression levels of KDR (*VEGFR2*), *ABCB1*, and *ESR2* were significantly decreased in the tumor tissues. No significant difference in *COL18A1* expression was observed between tumor and normal tissues.

Notably, the increased expression of mevalonate pathway–related genes GGPS1 and FDPS suggests that this metabolic pathway may play an active role in breast cancer biology. Furthermore, the low hazard ratio values observed for *ESR1* and *ESR2* support the notion that hormone receptor–related mechanisms are associated with more favorable clinical outcomes. In contrast, the association between elevated *FDPS*, *MMP9*, and *BRCA2* expression and poor prognosis indicates that these genes may serve as potential adverse prognostic biomarkers in breast cancer. Overall, the findings suggest that molecular mechanisms related to the mevalonate pathway, hormone receptor signaling, extracellular matrix remodeling, angiogenesis, and *RANK*/*RANKL*/*OPG* axis may play important roles in breast cancer progression.

In the present study, integrated network, expression, and survival analyses identified a biologically interconnected molecular signature involving the mevalonate pathway, hormone receptor signaling, angiogenesis, extracellular matrix remodeling, and the *RANK*/*RANKL*/*OPG* axis in breast cancer. STRING and GeneMANIA analyses demonstrated that the investigated genes form a highly interconnected network with significantly greater interactions than expected by chance, supporting their participation in common biological processes relevant to tumor progression. The central positioning of *ESR1*, *ESR2*, *KDR*, *MMP9*, *TNFSF11*, *TNFRSF11B*, and *COL18A1* within the interaction networks suggests that these genes may act as key regulators of breast cancer development and progression.

One of the most notable findings was the increased expression of FDPS and GGPS1, two critical enzymes in the mevalonate pathway. Dysregulation of the mevalonate pathway has emerged as an important hallmark of cancer metabolism, promoting cellular proliferation, migration, and resistance to apoptosis [57,58]. Bisphosphonates, particularly nitrogen-containing compounds such as alendronate and zoledronic acid, exert their pharmacological effects primarily by inhibiting *FDPS* and downstream prenylation processes [59]. Increased *FDPS* expression and its association with poor survival in our study support previous evidence indicating that activation of the mevalonate pathway contributes to aggressive tumor behavior and unfavorable clinical outcomes [60].

The observed overexpression of *MMP9* in breast cancer tissues and its association with reduced overall survival are consistent with previous studies demonstrating the role of MMP9 in extracellular matrix degradation, tumor invasion, and metastatic dissemination [61,62]. MMP9-mediated remodeling of the tumor microenvironment facilitates angiogenesis and promotes metastatic spread, particularly to the bone, which represents a major complication of advanced breast cancer. The identification of MMP9 as a poor prognostic marker further supports its potential as a therapeutic target for HCC.

Another important finding was the differential behavior of the estrogen receptor genes. Although ESR1 expression was significantly increased in tumor tissues, higher ESR1 expression was associated with better survival rates. This observation agrees with previous reports indicating that ER-positive breast cancers generally exhibit more favorable clinical outcomes and respond better to endocrine therapies than receptor-negative tumors [63,64]. Similarly, elevated ESR2 expression was associated with improved survival, supporting the evidence that ERβ may exert anti-proliferative and tumor-suppressive effects in breast cancer [64]. The central role of ESR1 and ESR2 in the interaction network also highlights the importance of hormone-dependent signaling pathways in coordinating multiple biological processes involved in breast cancer progression.

The present study further demonstrated significant alterations in the *RANK*/*RANKL*/*OPG* signaling axis. Increased *TNFSF11* (*RANKL*) expression, along with favorable survival associations for both *RANKL* and *OPG*, may initially appear paradoxical. However, previous studies have shown that the biological effects of the *RANK* pathway are highly context-dependent and influenced by interactions with hormonal signaling and immune regulation [65,66]. The *RANK*/*RANKL* pathway plays a critical role in mammary gland biology and bone metastasis formation, making it an attractive target for therapeutic interventions. These findings are particularly relevant because denosumab, a monoclonal antibody targeting *RANKL*, has shown beneficial effects in patients with breast cancer with bone involvement [67].

The reduced expression of *KDR* (*VEGFR2*) observed in tumor tissues contrasts with the established role of VEGF signaling in angiogenesis. Nevertheless, elevated *KDR* expression was associated with improved survival, suggesting that VEGF-related signaling may have subtype-specific effects in breast cancer. Previous studies have demonstrated complex interactions between VEGF receptors, vascular normalization, and treatment responses, which may partly explain these findings [68]. The GeneMANIA network also identified VEGFC as a closely related gene, further emphasizing the relevance of angiogenic pathways.

Interestingly, *ABCB1* expression was decreased in tumor tissues, whereas higher expression was correlated with better survival. Although *ABCB1* is commonly associated with multidrug resistance, recent evidence suggests that its prognostic significance varies among breast cancer subtypes and may reflect broader alterations in cellular transport and metabolic adaptation mechanisms [69]. This finding highlights the complexity of interpreting *ABCB1* biology based solely on its traditional role in chemotherapy resistance.

The increased expression and poor prognostic significance of *BRCA2* observed in the present study may reflect enhanced DNA damage response and genomic instability within aggressive tumors. Although *BRCA2* is traditionally regarded as a tumor suppressor gene, its elevated expression in established tumors has been associated with increased proliferative activity and adverse clinical characteristics in certain molecular subtypes [70]. These findings warrant further investigation into the context-dependent role of *BRCA2* expression in breast cancer.

Collectively, the current results suggest that mevalonate pathway activation, extracellular matrix remodeling, hormone receptor signaling, angiogenesis, and the *RANK*/*RANKL*/*OPG* axis represent interconnected biological processes that contribute to breast cancer progression. The identification of *FDPS* and *GGPS1* as overexpressed components of the mevalonate pathway provides additional mechanistic support for the potential repurposing of bisphosphonates in the management of breast cancer. Furthermore, the integration of network analyses, gene expression profiling, and survival data strengthens the rationale for investigating these pathways as therapeutic targets and prognostic biomarkers in future experimental and clinical studies.

## 4. Conclusions

This study provides an integrated computational assessment of alendronate, risedronate, and zoledronate using DFT, ADMET, molecular docking, and bioinformatics analyses. DFT calculations demonstrated distinct electronic properties among the investigated bisphosphonates, with zoledronate exhibiting the highest chemical reactivity and risedronate exhibiting the greatest kinetic stability. ADMET analyses indicated acceptable pharmacokinetic and safety profiles, although their high polarity may limit their oral bioavailability. Molecular docking analyses revealed favorable interactions with multiple proteins involved in breast cancer progression and bone remodeling. Zoledronate generally exhibited the strongest binding affinities, particularly toward *ESR2*, *VEGFR*/*KDR*, *GGPS1*, and *FPPS*, whereas risedronate exhibited notable interactions with BRCA2 and MMP9. Bioinformatics analyses further demonstrated the significant dysregulation of *GGPS1*, *FDPS*, *TNFSF11*, *ESR1*, *MMP9*, and *BRCA2* in breast cancer tissues and identified significant associations between several target genes and patient survival. STRING and GeneMANIA analyses highlighted strong functional interactions among the investigated genes and emphasized the involvement of the mevalonate pathway, hormone receptor signaling, angiogenesis, extracellular matrix remodeling, and the *RANK*/*RANKL*/*OPG* axis in breast cancer progression. Overall, these findings suggest that bisphosphonates, particularly zoledronate, may exert biological effects beyond bone resorption inhibition by modulating the molecular pathways associated with breast cancer. Further experimental and clinical studies are required to validate these computational findings and clarify their therapeutic potential. Despite the promising computational results, the present study has certain limitations inherent to in silico approaches. Therefore, future investigations should focus on validating the predicted molecular interactions and biological activities through in vitro and in vivo experiments. In addition, the high polarity and limited oral bioavailability predicted for the investigated bisphosphonates suggest that structural optimization may further enhance their therapeutic potential. The design and evaluation of novel bisphosphonate analogues with improved pharmacokinetic properties, while preserving their favorable target-binding characteristics, may represent an important avenue for future drug development in breast cancer-associated bone disease.

## 5. Limitations

This study had some limitations that should be acknowledged. First, the findings were generated entirely through computational approaches, including bioinformatics analyses, molecular docking, ADMET predictions, and DFT calculations; therefore, they require experimental validation. Second, molecular docking analyses were performed using static protein structures, which may not fully capture protein flexibility, molecular dynamics, or the complexity of the tumor microenvironment. Third, the bioinformatics analyses relied on publicly available databases (e.g., TCGA and GTEx). While these are robust resources, they inherently carry potential biases, including demographic imbalances, sampling biases, and incomplete clinical annotations. Consequently, the potential influences of patient heterogeneity, varying molecular subtypes, prior treatment histories, and latent clinical characteristics could not be comprehensively evaluated. Furthermore, the gene expression results were not validated at the protein level or through functional experiments. Despite these limitations, the integration of bioinformatics, docking, ADMET, and DFT analyses provides a comprehensive preliminary assessment of the potential molecular effects of bisphosphonates in breast cancer and offers a basis for future experimental and clinical studies.

## Figures and Tables

**Table 1:** HOMO–LUMO energies and chemical parameters of the compounds studied (Alendronate, Risedronate and Zoledronate) calculated by B3LYP.

Compounds	E_HOMO_	E_HOMO−1_	E_LUMO_	E_LUMO+1_	ΔE	*I*	*A*	*η*	*s*	*μ*	*χ*	ω
Alendronate	−6.1076	−6.2369	−0.9449	−1.8745	5.1627	6.1076	0.9449	2.5814	0.1937	−3.5263	3.5263	22.8308
Risedronate	−6.8809	−6.8848	−0.1341	−1.7603	6.7468	6.8809	0.1341	3.3734	0.1482	−3.5075	3.5075	1.8236
Zoledronate	−5.8771	−6.4434	−2.9102	−0.9736	2.9669	5.8771	2.9102	1.4835	0.3370	−4.3937	4.3937	2.4097

ΔE → Energy Gap, *I* → Ionization Potential (−E_HOMO_), *A* → Electron Affinity (−E_LUMO_), *η* → Chemical hardness, *s* → Chemical softness, *μ* → Chemical Potential, *χ* → Electronegativity, ω → Electrophilicity index.

**Table 2:** Calculated NLO parameters of the investigated compounds using B3LYP.

Compounds	μ (Debye)	α (au)	β (esu)
Alendronate	6.1039	−97.0397	6.18 × 10^−30^
Risedronate	3.5672	−107.3774	3.57 × 10^−30^
Zoledronate	8.7051	−96.7631	1.20 × 10^−30^

Urea (Reference) = μ_(D)_ = 1.3197, β (esu) = 0.1947 × 10^−30^.

**Table 3:** The Alendronate, Risedronate and Zoledronate compounds’ physicochemical, lipophilicity and ADMET parameters.

Property	Alendronate	Risedronate	Zoledronate
Molecular Weight	249.02	282.01	272.0
nHA	8	7	9
nHD	7	5	5
logP	−3.082	−0.194	−1.725
TPSA	161.31	135.29	153.11
HIA	0.999	0.999	1.0
Caco-2 Permeability	6.23	−6.196	−6.329
BBB	0.095	0.397	0.14
PPB	17.62%	42.35%	20.52%
VD	0.682	0.34	0.589
CYP2D6	0.211	0.231	0.117
CYP3A4	0.007	0.013	0.009
T_1/2_	0.159	0.184	0.221
hERG Blockers	0.037	0.044	0.038
H-HT	0.044	0.03	0.162
AMES Toxicity	0.04	0.011	0.058
Rat Oral Acute Toxicity	0.0	0.001	0.058

**Table 4:** Docking scores of the Alendronate, Risedronate and Zoledronate against enzymes of the study.

Docking Score (kcal/mol)	Compounds		
	Alendronate	Risedronate	Zoledronate
PDB: 1A52	−5.70	−6.70	−5.50
PDB: 2FSZ	−7.20	−7.00	−7.80
PDB: 1MIU	−5.30	−7.70	−7.00
PDB: 1GKC	−7.40	−7.70	−7.00
PDB: 6QEX	−5.40	−7.50	−7.10
PDB: 3VHE	−7.40	−7.70	−7.60
PDB: 1BNL	−6.30	−6.50	−7.70
PDB: 4E4D	−6.20	−6.40	−6.30
PDB: 3URF	−7.00	−7.30	−7.40
PDB: 2Q80	−7.10	−7.40	−7.50
PDB: 2F8C	−7.20	−7.30	−7.30

**Table 5:** The interaction parameters between the Alendronate, compound and PDB: 2F8C, PDB: 2Q80 and PDB: 3VHE enzymes.

Type of Bond	Alendronate PDB: 2F8C	Bond Length (Å)	Alendronate PDB: 2Q80	Bond Length (Å)	Alendronate PDB: 3VHE	Bond Length (Å)
Van der Waals Interactions	PRO-179 ILE-105 - - - - -	- - - - - - -	LYS-202 GLN-185 THR-152 LEU-155 HIS-57 GLN-126 LEU-61	- - - - - - -	CYS-1024 LEU-1019 LYS-868 ILE-888 ILE-892 - -	- - - - - - -
Conventional Hydrogen Bonds	LEU-175 MET-106 SER-108 ALA-178	3.73 5.33 4.02 3.24, 4.83	LYS-151 ARG-73 ASP-68 ASP-64	4.75 5.32, 5.49 7.20 5.17, 5.52	HIS-1026 ILE-1025 - -	3.06 4.13 - -
Attractive Charge	ASP-107 ASP-264 - -	5.43 4.49 - -	ASP-68 ASP-129 ASP-188 ASP-64	7.10 7.62 6.23, 7.35 5.75, 6.42	ASP-814 ASP-1046 - -	5.76 4.78, 7.17 - -
Carbon Hydrogen Bond	- -	- -	LYS-212 -	5.53 -	HIS-1026 ASP-1046	3.71 4.01
Un-favorable Donor-Donor	LYS-266 GLN-180	4.55 4.04	LYS-212 -	5.40 -	ARG-1027 -	4.32 -
Pi-Cation	-	-	-	-	HIS-1026	3.06
Un-favorable Acceptor-Acceptor	MET-106	5.81	-	-	ILE-1025	5.74, 5.76
Un-favorable Positive-Positive	LYS-266	7.40	-	-	-	-
Pi-Anion	-	-	-	-	GLU-885	4.52
Pi-Alkyl	-	-	LYS-151	4.75	-	-

**Table 6:** The interaction parameters between the Risedronate compound and PDB: 1MIU, PDB: 1GKC and PDB: 3VHE enzymes.

Type of Bond	Risedronate PDB: 1MIU	Bond Length (Å)	Risedronate PDB: 1GKC	Bond Length (Å)	Risedronate PDB: 3VHE	Bond Length (Å)
Van der Waals Interactions	GLN-2969 PRO-2970 PRO-2986 VAL-2991 ASP-2992 SER-2973 -	- - - - - - -	GLY-186 LEU-187 HIS-190 LEU-418 TYR-420 PRO-421 TYR-423	- - - - - - -	LYS-1039 LEU-896 ARG-863 GLU-917 LYS-920 THR-864 CYS-919	- - - - - - -
Conventional Hydrogen Bonds	GLU-2972	2.76, 4.09 4.90	ALA-189	2.89, 3.63, 4.23	LYS-1043 SER-1037 PHE-918	5.88 4.30, 5.04 4.37
Attractive Charge	GLU-2972	4.45, 4.65	GLU-402	6.45	GLU-1038	4.62
Carbon Hydrogen Bond	GLU-2990 ARG-297	3.96 4.58	MET-422 -	4.41 -	- -	5.84 4.51
Unfavorable Donor-Donor	LEU-2974 AER-2978	4.14 4.89	LEU-188 -	3.88 -	- -	- -
Pi-Cation	ARG-2745	6.66	-	-		
Pi-Alkyl	PRO-2987 ARG-2971	5.35 5.18	VAL-398 -	5.29 -	VAL-1041 -	6.40 -
Pi-Pi Stacked	-	-	HIS-401	5.42		
Un-favorable Acceptor-Acceptor	-	-	-	-	GLU-1038	4.81
Pi-Donor Hydrogen Bond	-	-	-	-	SER-1037	5.59

**Table 7:** The interaction parameters between Zoledronate compound and PDB: 2FSZ, PDB: 1BNL, and PDB: 3VHE enzymes.

Type of Bond	Zoledronate PDB: 2FSZ	Bond Length (Å)	Zoledronate PDB: 1BNL	Bond Length (Å)	Zoledronate PDB: 3VHE	Bond Length (Å)
Van der Waals Interactions	TRP-345 HIS-308 VAL-338 VAL-280 LEU-339	- - - - -	GLN-35 GLN-36 - - -	- - - - -	ILE-888 ILE-892 CYS-1024 LEU-1019 LYS-868	- - - - -
Conventional Hydrogen Bonds	LYS-401 TYR-397 GLU-305 HIS-279 PRO-278 PRO-278 ARG-346	6.56 5.27 5.40 7.41 4.40 5.64 3.94, 4.48 6.64	ASN-16 ARG-38 - - - - - -	5.24 3.76 - - - - - -	ILE-1025 HIS-1026 - - - - - -	4.13 3.71 - - - - - -
Attractive Charge	GLU-276 GLU-305	6.60 3.78, 5.81	ASP-104	5.55	ASP-814 ASP-1046	5.76 4.78, 7.17
Carbon Hydrogen Bond	GLY-342 HIS-279	3.66 3.09	GLN-35 -	4.26 -	HIS-1026 ASP-1046	3.06 4.01
Un-favorable Donor-Donor	-	-	GLN-35	4.54	ARG-1027	4.32
Pi-Cation	HIS-279	3.30	-	-	HIS-1026	4.29
Un-favorable Acceptor-Acceptor	PRO-278	4.45	ASP-104	4.13	ILE-1025	5.76
Pi-Anion	GLU-305	5.81	-	-	GLU-885	4.52
Pi-Sulfur	MET-309	5.31	-	-	-	-
Pi-Alkyl	PRO-277	4.21	ALA-39	5.25, 5.34	-	-
Pi-Sigma	-	-	VAL-40	6.09	-	-

## Data Availability

The original contributions presented in this study are included in the article. Further inquiries can be directed to the corresponding author.

## Supplementary Materials

The following supporting information can be downloaded at https://www.mdpi.com/article/10.3390/biology15120952/s1: Figure S1: Two-dimensional (2D) interaction diagrams of Alendronate with the selected protein targets of the study. Figure S2: Two-dimensional (2D) interaction diagrams of risedronate with the selected protein targets of the study. Figure S3: Two-dimensional (2D) interaction diagrams of Zoledronate with the selected protein targets of the study. Table S1: Calculated nonlinear optical (NLO) parameters of alendronate obtained at the B3LYP level of theory. Table S2: Calculated nonlinear optical (NLO) parameters of risedronate obtained at the B3LYP level of theory. Table S3: Calculated nonlinear optical (NLO) parameters of zoledronate obtained at the B3LYP level of theory.

## Abbreviations

The following abbreviations are used in this manuscript:

ABCB1	ATP-Binding Cassette Subfamily B Member 1
ADMET	Absorption, Distribution, Metabolism, Excretion, and Toxicity
BBB	Blood–Brain Barrier
BRCA2	Breast Cancer Type 2 Susceptibility Protein
DFT	Density Functional Theory
ESR1	Estrogen Receptor Alpha
ESR2	Estrogen Receptor Beta
FDPS (FPPS)	Farnesyl Diphosphate Synthase
FMO	Frontier Molecular Orbital
GGPS1	Geranylgeranyl Diphosphate Synthase 1
GTEx	Genotype-Tissue Expression
HIA	Human Intestinal Absorption
HOMO	Highest Occupied Molecular Orbital
KDR (VEGFR2)	Kinase Insert Domain Receptor (Vascular Endothelial Growth Factor Receptor 2)
LUMO	Lowest Unoccupied Molecular Orbital
MEP	Molecular Electrostatic Potential
MMP9	Matrix Metalloproteinase-9
NLO	Nonlinear Optical
OPG (TNFRSF11B)	Osteoprotegerin
PDB	Protein Data Bank
PPI	Protein–Protein Interaction
PPB	Plasma Protein Binding
RANKL (TNFSF11)	Receptor Activator of Nuclear Factor Kappa-B Ligand
SHG	Second Harmonic Generation
STRING	Search Tool for the Retrieval of Interacting Genes/Proteins
TCGA	The Cancer Genome Atlas
TPSA	Topological Polar Surface Area
VD	Volume of Distribution
VEGFR	Vascular Endothelial Growth Factor Receptor
