# Supplementary material for: The Effects of Bisphosphonates Used in Osteoporosis Treatment on Breast Cancer: Analysis with Integrative Bioinformatics Methods, DFT, ADMET and Molecular Docking Analysis
Source: Biology (Basel). 2026 Jun 18;15(12):952. doi: 10.3390/biology15120952 (PMC13296182; doi:10.3390/biology15120952)
Supplement: Supplementary file 1 [file biology-15-00952-s001.zip › biology-4380606-supplementary.pdf]

Article Title: The Effects of Bisphosphonates Used in Osteoporosis Treatment on Breast Cancer: Analysis with Integrative Bioinformatics Methods, DFT, ADMET and Molecular Docking Analysis

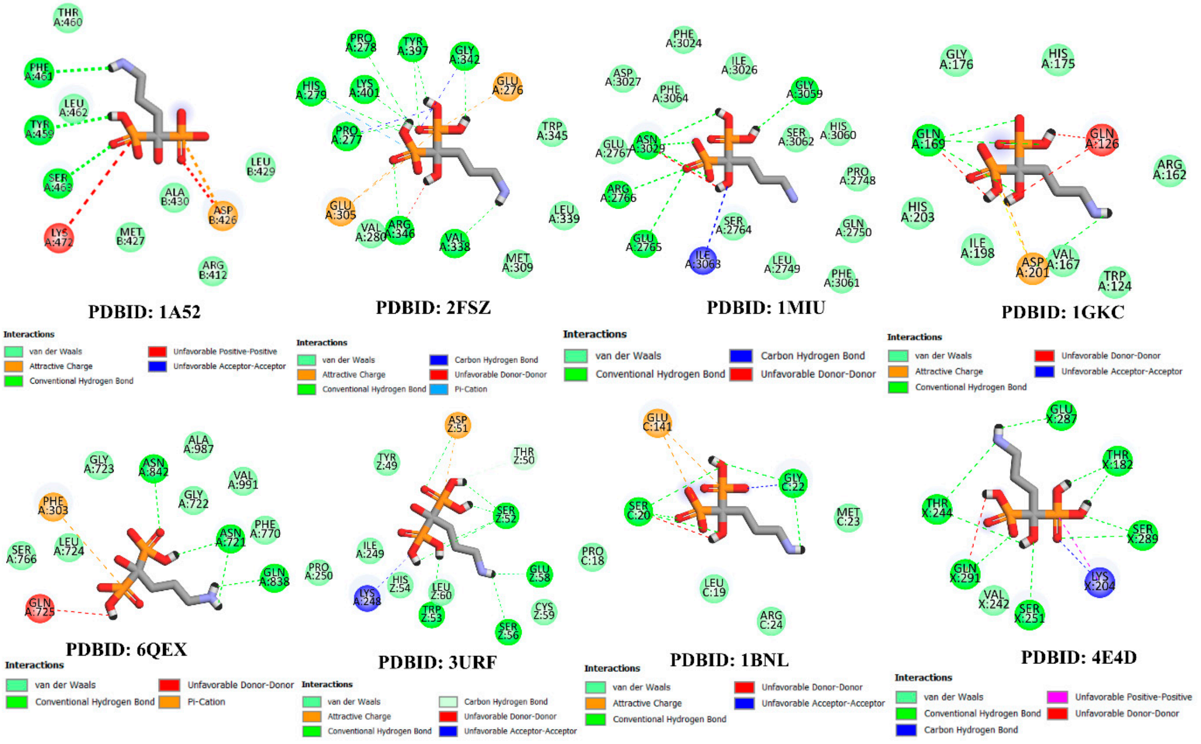

Figure S1. Two-dimensional (2D) interaction diagrams of Alendronate with the selected protein targets of the study.

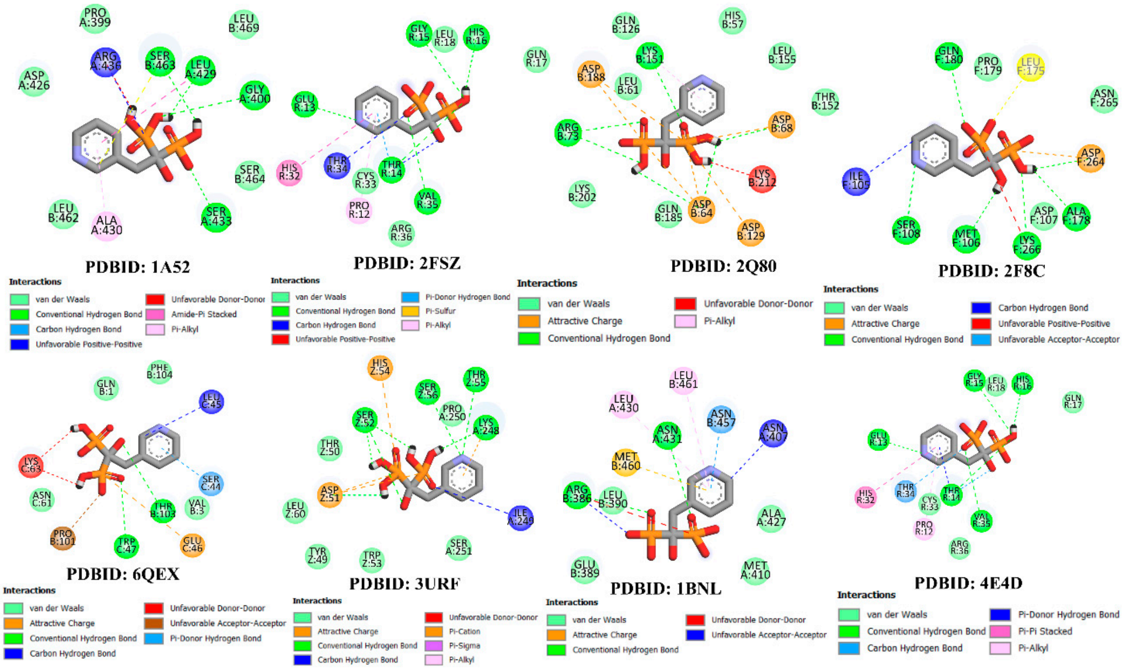

Figure S2. Two-dimensional (2D) interaction diagrams of risedronate with the selected protein targets of the study.

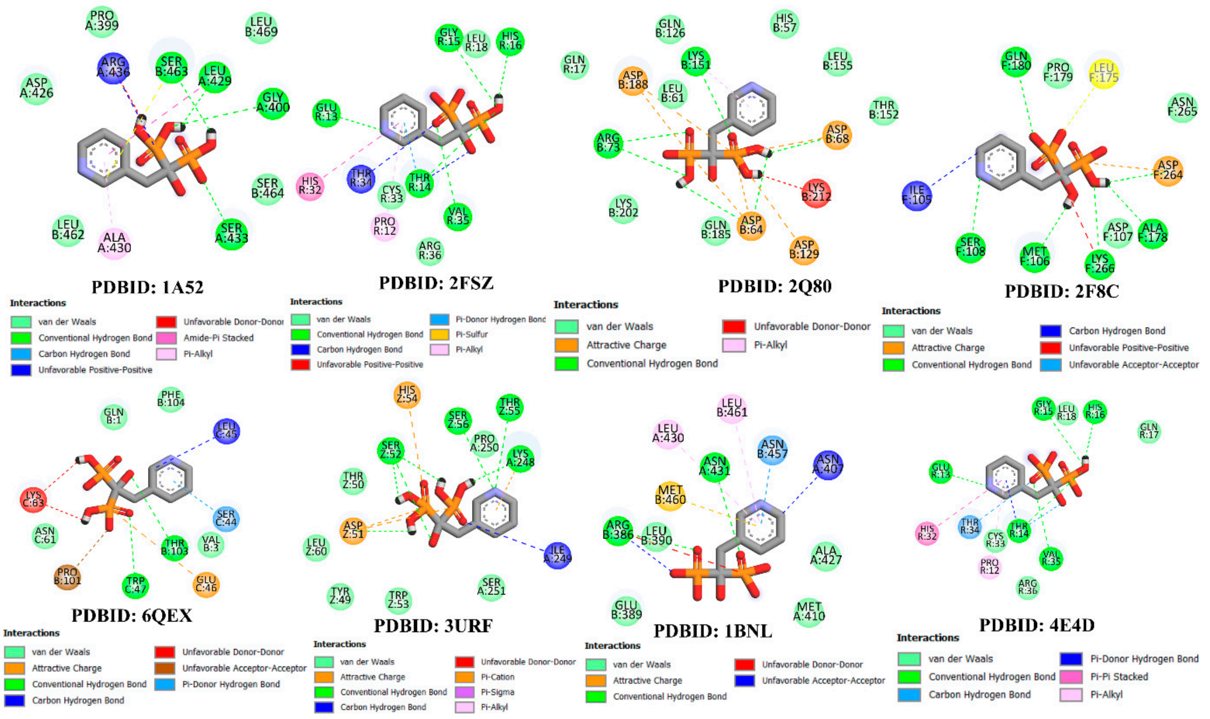

Figure S3. Two-dimensional (2D) interaction diagrams of Zoledronate with the selected protein targets of the study.

Table S1. Calculated NLO parameters of Alendronate using B3LYP.

Parameters	Alendronate	Parameters	Alendronate
μ_x	3.6441	β_{XXX}	8.0376
μ_y	3.2815	β_{YYY}	34.4785
μ_z	3.6345	β_{ZZZ}	12.0837
$\mu_{(D)}$	6.1039	β_{XYY}	-8.5872
α_{XX}	-100.4409	β_{XXY}	24.0722
α_{YY}	-97.8981	β_{XXZ}	9.7512
α_{ZZ}	-92.7801	β_{XZZ}	-2.5690
α_{XY}	5.9343	β_{YZZ}	0.1902
α_{XZ}	7.7325	β_{YYZ}	18.8947
α_{YZ}	7.9732	β_{XYZ}	-25.9968
$\alpha_{(au)}$	−97.0397	$\beta_{(esu)}$	6.18×10^{-31}

Table S2. Calculated NLO parameters of Risedronate using B3LYP.

Parameters	Risedronate	Parameters	Risedronate
μ_x	1.3229	β_{XXX}	-20.7679
μ_y	-0.5074	β_{YYY}	-21.6607
μ_z	3.2737	β_{ZZZ}	17.5296
$\mu_{(D)}$	3.5672	β_{XYY}	5.6430
α_{XX}	-94.1834	β_{XXY}	0.3122
α_{YY}	-114.7413	β_{XXZ}	6.5349
α_{ZZ}	-113.2074	β_{XZZ}	-17.0821
α_{XY}	-15.3660	β_{YZZ}	8.6775

α_{XZ}	0.2372	β_{YYZ}	-1.7805
α_{YZ}	-4.5581	β_{XYZ}	3.4706
$\alpha_{(au)}$	−107.3774	$\beta_{(esu)}$	3.57×10^{-31}

Table S3. Calculated NLO parameters of Zoledronate using B3LYP.

Parameters	Zoledronate	Parameters	Zoledronate
μ_x	-7.9134	β_{XXX}	-96.6158
μ_y	-1.8242	β_{YYY}	23.7723
μ_z	3.1353	β_{ZZZ}	17.8200
$\mu_{(D)}$	8.7051	β_{XYY}	-17.1502
αXX	-67.3090	β_{XXY}	-60.1907
α_{YY}	-118.1942	β_{XXZ}	14.1855
αZZ	-104.7862	β_{XZZ}	-10.8748
α_{XY}	-5.6378	β_{YZZ}	-1.8246
α_{XZ}	1.9857	β_{YYZ}	17.2527
α_{YZ}	-15.5402	β_{XYZ}	4.9361
$\alpha_{(au)}$	-96.7631	$\beta_{(esu)}$	1.20×10^{-30}
